# Cross-Talk-Free Multi-Color STORM Imaging Using a Single Fluorophore

**DOI:** 10.1371/journal.pone.0101772

**Published:** 2014-07-07

**Authors:** Johnny Tam, Guillaume Alan Cordier, Joseph Steven Borbely, Ángel Sandoval Álvarez, Melike Lakadamyali

**Affiliations:** ICFO-Institut de Ciències Fotòniques, Mediterranean Technology Park, Castelledefels, Barcelona, Spain; University of New South Wales, Australia

## Abstract

Multi-color stochastic optical reconstruction microscopy (STORM) is routinely performed; however, the various approaches for achieving multiple colors have important caveats. Color cross-talk, limited availability of spectrally distinct fluorophores with optimal brightness and duty cycle, incompatibility of imaging buffers for different fluorophores, and chromatic aberrations impact the spatial resolution and ultimately the number of colors that can be achieved. We overcome these complexities and develop a simple approach for multi-color STORM imaging using a single fluorophore and sequential labelling. In addition, we present a simple and versatile method to locate the same region of interest on different days and even on different microscopes. In combination, these approaches enable cross-talk-free multi-color imaging of sub-cellular structures.

## Introduction

Stochastic optical reconstruction microscopy (STORM) [1] and similar methods (including photoactivated localization microscopy, PALM, and fluorescence photoactivated localization microscopy, fPALM) [2], [3] enable fluorescence imaging beyond the diffraction limit, extending the spatial resolution of optical microscopy to nanometer length scales. STORM imaging relies on two important concepts. First, the position of a single fluorescent molecule can be precisely determined if its image is isolated in space [4], [5]. Second, photoswitchable fluorophores [6]–[9] can be used to overcome the problem that when multiple fluorescent molecules overlap in a diffraction limited volume, their images merge, making it difficult to determine their positions. By switching most of the fluorescent molecules into a dark state and photoactivating only a sparse subset of them, it is possible to obtain isolated images of single molecules and localize their positions precisely. By repeating the photoactivation, imaging and localization, a high resolution image of the underlying structure can be reconstructed from fluorophore positions.

Recent years have seen a tremendous amount of technological development in single molecule based super-resolution microscopy methods such as STORM [10]–[22]. Multi-color imaging is an important capability of fluorescence microscopy since it allows for a determination of colocalization and interaction between different sub-cellular structures. STORM imaging was extended to multiple colors soon after its initial discovery [23], [24]. However, the various approaches used for multi-color STORM imaging have important caveats. These caveats lead to decreased resolution and increased complexity as the number of colors is increased. One approach for multi-color STORM uses fluorophore pairs in which the same reporter is coupled to different activators [23]. In this case, the color is determined based on the wavelength of the activating laser light. By using pulses of activation laser light with different wavelengths, it is possible to color-code the resulting localizations based on when they turn on during the imaging cycle [23], [25]. This approach is free from chromatic aberrations and the need for image registration since all colors are acquired in the same image channel. However, it is prone to color cross-talk [24], since fluorophores can also undergo spontaneous activation, independent of the activation pulse, or alternately, fluorophores can be activated by the “wrong” activation pulse. A second approach uses spectrally-distinct reporter dyes coupled to the same (or different) activator dyes [24]. A variation of this second approach also exists that uses spectrally-distinct photoswitchable reporter fluorophores alone without an activator dye (dSTORM) [26]–[29]. The advantage of these approaches is that color cross-talk can be reduced or eliminated. However, chromatic aberrations can be difficult to correct at the nanoscale level [30]. More importantly, there is a limited availability of spectrally distinct photoswitchable fluorophores with favourable photophysical properties. Differences in the duty cycle and brightness of different fluorophores can impact the relative resolution of the images in the different color channels. In particular, the best fluorophore can only be used once. A detailed analysis of a large number of photoswitchable fluorophores showed that AlexaFluor647 outperforms most fluorophores leading to images with the highest resolution [26]. Finally, it is often difficult to find one optimal imaging buffer compatible with all fluorophores. The performance of the imaging buffer can also decrease over time as more colors are acquired.

Here, we show that all these caveats can be overcome with a multi-color STORM imaging approach that uses the same fluorophore for all the colors. This approach is based on sequential labelling and STORM imaging and utilizes a simple and versatile strategy that enables the same region to be located for different imaging sessions and even for different microscopes. Sequential labelling has previously been shown to be useful at the conventional fluorescence level for imaging several different protein species [31]. At the STORM level, this approach enables us to always use the best imaging buffer conditions and the best-performing fluorophore for STORM, without any cross-talk or need to consider chromatic aberrations.

## Results

We demonstrate a strategy for multi-color STORM imaging based on correlative microscopy, in which the same sample is repeatedly imaged using different modalities –instead of varying the modality, we vary the color, and perform sequential STORM imaging. Each STORM imaging session is carried out under identical imaging conditions (i.e. with the same fluorophore and imaging buffer), eliminating variations in spatial resolution due to fluorophore performance.

### Sequential imaging using a virtual grid to repeatedly locate a given region of interest

Sequential or correlative imaging requires that the same region of interest be located on multiple imaging sessions or microscopes. Otherwise, carrying out the immunostaining *in situ* on the microscope stage in between imaging sessions will lead to long periods of “down time” during which the microscope is not acquiring data. As the number of colors to be imaged increases, the time that the sample must remain on the microscope stage also increases, rendering the microscope unusable for other experiments and decreasing efficiency. To limit the microscope down time and to increase the flexibility and ease of imaging, we developed a simple and versatile approach we termed “virtual grid” to repeatedly and reliably locate the same region of interest on different imaging sessions and even on different microscopes.

**Figure 1:**
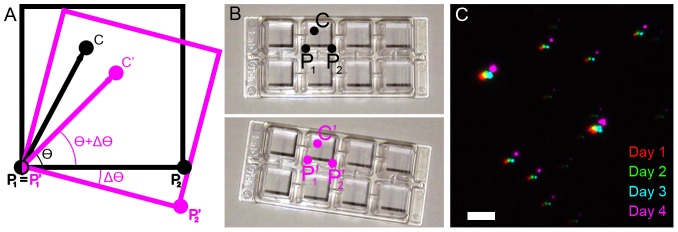
Virtual grid to relocate the same region of interest. (A–B) During the first imaging session, the coordinates of two reference points are recorded (*P_1_* and *P_2_*, typically the corner coordinates of the sample chamber as shown in B) as well as the coordinates of the region of interest, *C*. During the subsequent imaging sessions, the new coordinates of the reference points are recorded (*P_1_'* and *P_2_'*) and these coordinates along with the previously recorded coordinates of the reference points and region of interest are used to calculate the new coordinates of the region of interest (*C*′). (C) Fiduciary markers (fluorescent beads) imaged on four subsequent days using the “virtual grid” approach to locate them. Scale bar 5 µm.

The use of high magnification objectives in STORM imaging implies that the field of view being imaged is relatively small (typically 40×40 µm^2^ or smaller). Therefore, finding the same region can be highly challenging once the sample is removed from the microscope. In correlative microscopy, a “finder grid” is used for this purpose [32]. However, glass-bottom chambers that are readily available and used by most laboratories for fluorescence microscopy applications typically do not include a finder grid. It would be beneficial to most people working with fluorescence microscopy to have a simple method for finding the same region of interest on multiple days and even multiple microscopes without the need for a physical grid. Here, we demonstrate a “virtual grid” which functions like a grid but without the need for a physical grid (**Fig 1a**). The virtual grid can be implemented using a precision motorized stage with a readout for the stage coordinates in the *X* and *Y* directions. During the first imaging session, the coordinates were recorded of the region that was imaged, *C*, along with the coordinates of two reference points, which were defined as the two corner points of the sample chamber, *P_1_* and *P_2_* (**Fig 1a and 1b**). At the start of the next imaging session, the two reference points were relocated and the coordinates of these two points were recorded as *P_1_'* and *P_2_'*. These reference points were used to locate the cell. The rotation angle between day 1 and day 2 was calculated as:
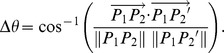



With careful mounting of the sample, we could maintain Δ*θ* below 0.05 degrees. The new location of the region, *C′*, relative to the corner point *P_1_'* was calculated as:
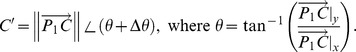



This procedure allowed us to locate the same region of interest within 5 µm (one field of view is approximately 40×40 µm in our microscope, and the precision of our motorized stage is around 2 µm). To locate the region more precisely, images of fiduciary markers (fluorescent beads) were acquired and compared to images of these same markers from the first imaging session. This enabled us to fine-tune the position of the stage (**Fig 1c**).

### Multi-color STORM imaging using the same fluorophore

**Figure 2:**
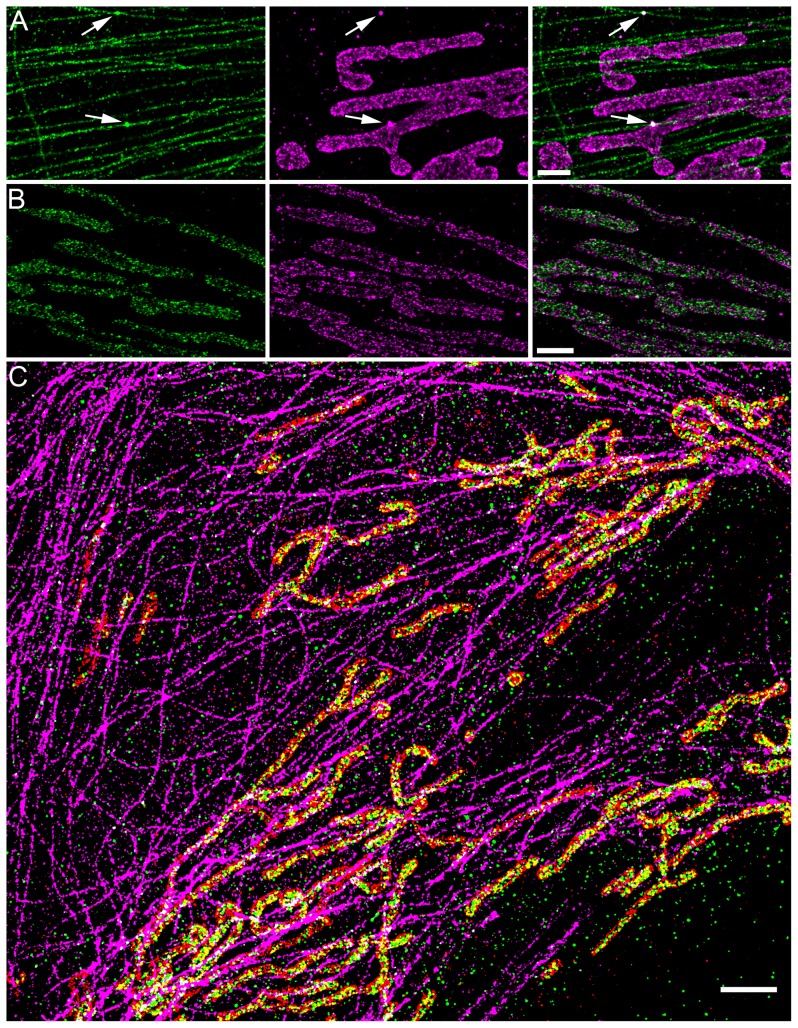
Multi-color STORM imaging using a single fluorophore. (A) Microtubules (green) and mitochondrial outer membrane protein Tom20 (magenta) imaged sequentially using the same fluorophore activator-reporter pair (AlexaFluor405-AlexaFluor647). Arrows show the localized positions of fiduciary markers (fluorescent beads) that were used for image alignment. (B) Mitochondrial outer membrane protein Tom20 (magenta) and inner membrane protein ATP Synthase (green). (C) Three-color image of microtubules (green), mitochondrial outer membrane protein Tom20 (magenta) and mitochondrial inner membrane protein (ATP-synthase, orange) imaged sequentially using the same fluorophore activator-reporter pair (AlexaFluor405-AlexaFluor647). The discontinuous appearance of microtubules is due to the fact that we have used an anti-GFP antibody to label the GFP-α-tubulin and the endogenous α-tubulin is unlabelled in this scheme. The anti-GFP antibody was used since it offers a different antibody species to those used for ATP-synthase and Tom20. Scale bars, 1 µm (A–B), and 2 µm (C).

To demonstrate multi-color STORM imaging using the same fluorophore, we labelled the first target structure of interest (microtubules in **Fig 2a**, green, and mitochondrial inner membrane in **Fig 2b**, green) with an appropriate primary antibody (anti-α-tubulin for microtubules and anti-ATP-synthase for mitochondrial inner membrane) followed by secondary antibody conjugated with a STORM compatible fluorophore pair (AlexaFluor405-AlexaFluor647). After recording a STORM image, the sample was removed from the microscope stage, the remaining (unbleached) fluorophores were quenched by adding a reducing agent (sodium borohydride, see Methods) and a second target structure (mitochondria outer membrane in both **Fig 2a** and **Fig 2b**, magenta) was labelled with an appropriate primary antibody (anti-Tom20) derived from a species different from the previously used primary antibody. The secondary antibody, once again, was conjugated with the same fluorophore pair. The same region of interest was located using the virtual grid approach described above and a new STORM image was recorded. During the first imaging session, we set the focal plane such that both the sample and the fiduciary markers (fluorescent beads) were in focus at the same time. During subsequent imaging sessions, we manually adjusted the focus such that the fiduciary markers were at the same focus. Finally, after image acquisition, the images of fiduciary markers were used to precisely align the images (Methods). This sequential labelling and imaging scheme could be repeated as many times as desired to increase the number of colors (see **Fig 2c** for a 3-color combination).

The image registration precision was quantified by determining the average distance between the transformed centroid positions of fiduciary markers in one image with the centroid positions of these in the other image (Methods, Equation 1). While we normally use the positions of all the fiduciary markers to calculate the transformation function, optimal registration could be achieved with a minimum of three fiduciary markers for a first order polynomial affine transformation and six fiduciary markers for a second order polynomial local weighted mean transformation, as long as the fiduciary markers chosen for the registration were located on opposite corners of the field of view (Methods). We therefore computed the registration error using this minimum number of fiduciary markers. The average registration error was 12.2+/−3.0 and 10.5+/−2.5 nm (mean +/− standard deviation, n = 9) (**Fig S1**) for the affine and local weighted mean transformations, respectively. The registration error between different combinations of multiple sequential images did not change when the same sample was repositioned many times (**Table S1**). In the case of images acquired in two separate channels (therefore containing chromatic aberrations), the registration error was 17.3+/−2.9 and 12.1+/−3.8 nm (n = 9) for affine and local weighted mean transformations, respectively (**Fig S1**). Therefore, while the chromatic aberrations can be accounted for with fiduciary markers, the registration error is more dependent on the complexity of the registration algorithm in the case when chromatic aberrations are present.

The efficiency by which the fluorophores were quenched after the first imaging round was quantified by drawing regions of interest around the structure imaged in the first round and determining the number of localizations per unit area within these regions of interest in the first as well as the sequential image (**Fig S2**). For a microtubule sample that was labelled at high density (1∶70 dilution of primary antibody, typically used in STORM imaging) and imaged in the first round, followed by labelling and imaging of mitochondria in the second round, the microtubule localization density was 20177+/−8200 µm^−2^ (mean +/− standard deviation, n = 4 cells) in the first image. This density dropped to 590+/−286 µm^−2^ when considering the same regions in the subsequent image, which was comparable to the background localization density (490+/−234 µm^−2^) of regions that excluded both microtubule and mitochondria structures. As a comparison, for a two-color image of microtubules and mitochondria recorded using activator-reporter pairs undergoing the same labelling and sample preparation conditions, the localization density of the microtubules was 3777+/−2394 µm^−2^ (n = 2 cells) in the microtubule channel. This density was lower than the density measured for sequential imaging because only the frames immediately after the activation frame were included in the analysis to minimize color cross-talk. This restriction resulted in a reduction in the number of frames analysed. In this case, the localization density of these same regions in the mitochondria channel was 2191+/−2027 µm^−2^, which was higher than the background density of regions excluding both microtubules and mitochondria (287+/−337 µm^−2^), indicating a large degree of crosstalk. Therefore, our method not only eliminates cross-talk, but also improves the efficiency of accumulating localizations in each channel by not having to discard frames to minimize cross-talk.

Finally, to verify that the additional immunostaining steps performed between imaging sessions did not affect the integrity of the sample, we investigated the effect of repeated sample preparation steps on the structure of microtubules (**Fig S3**). Sequential images of the same microtubule network imaged before and after five rounds of immunostaining steps aligned within the previously calculated registration error (**Fig S3a and b**) and no structural defects or changes to the microtubule architecture were visible. The beads that were used for the registration also aligned within the previously determined registration error, indicating that the beads did not shift their position during the sample preparation (**Fig S3c**). This result shows that both the sample and the fiduciary markers used for precise alignment were not affected by additional rounds of immunostaining when compared to our calculated registration error.

### Multi-color STORM imaging using overlapping antibody species

**Figure 3:**
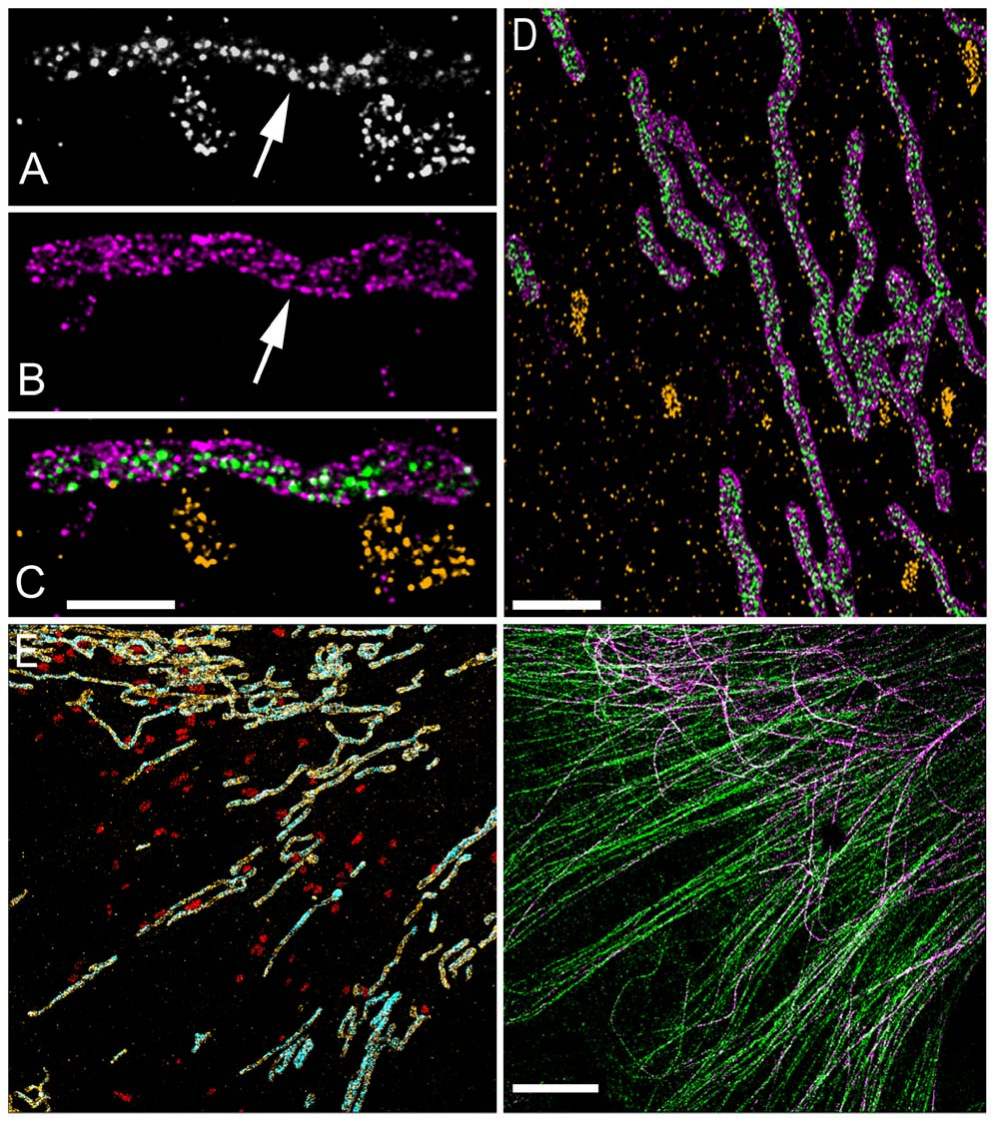
Multi-color STORM imaging using overlapping antibody species. (A) An image of ATP-synthase (localized to mitochondria) and LAMP2 (localized to lysosomes) both labelled using a mouse monoclonal primary and anti-mouse secondary antibody and imaged at the same time. (B) An image of Tom20, a mitochondrial outer membrane protein. Since Tom20 and ATP-synthase colocalize on mitochondria (arrows), the colocalization can be used to separate the initial image into separate colors. (C) ATP-synthase is identified as those molecules which colocalize with Tom20. Lysosomes are identified as those molecules which do not colocalize with Tom20. (D) A zoom-out of the three color Tom20 (magenta), ATP-Synthase (green), lysosome (orange) STORM image. (E) A five-color STORM image of mitochondrial outer membrane protein Tom20 (orange), mitochondrial inner membrane protein ATP-synthase (cyan), lysosomal protein Lamp2 (red), total tubulin (green) and acetylated tubulin (magenta). The five-color image is split between the two panels to more clearly display the different structures. The acetylated tubulin, ATP-synthase, and Lamp2 are all imaged using mouse primary antibodies. The acetylated tubulin colocalizes with total tubulin and ATP-synthase colocalizes with Tom20; Lamp2 does not colocalize with either total tubulin nor Tom20. Scale bars, 500 nm (C), 2 µm (D) and 5 µm (E).

Using our approach, multi-color STORM imaging should only be limited by the availability of well-performing primary antibodies derived from different species. However, sometimes the best-performing and most specific antibodies are monoclonal antibodies derived from the same species (e.g. mouse) and it may become difficult to avoid using two antibodies derived from the same species. In this case, one approach for multi-color imaging would be to directly label the primary antibody with STORM-compatible fluorophores followed by the sequential imaging approach as described above. However, fluorophore labelling of primary antibodies leads to a decrease in antibody concentration, and a decreased labelling density due to a lack of amplification from secondary antibodies. Optimization of primary antibody labelling can be very costly. Moreover, the common presence of other proteins inside the primary antibody buffer solution (such as BSA or ascites), which would also get labelled alongside the antibody, hinders the determination of the labelling efficiency and can lead to non-specific background in the images. To alleviate these problems and to potentially increase the number of colors that can be imaged in one sample, we borrowed an approach used in Electron Microscopy (EM) in which the images are grayscale but can be segmented into different colors due to the high spatial resolution offered by EM [33]. We demonstrate that multiple targets can be imaged using the same antibody species and that colors can be segmented based on the spatial separation of the different targets in the high resolution image in combination with the molecular specificity afforded by fluorescence microscopy. To demonstrate this point, as an example, we first recorded an image of ATP-synthase (localized to mitochondria) and LAMP2 (localized to lysosomes), both labelled using a mouse monoclonal primary and anti-mouse secondary antibody and imaged at the same time (**Fig 3a**). The high resolution of the final image led to spatial separation between mitochondria-like and lysosome-like structures. However, in some cases, the identity of a structure can be unclear simply from visual inspection. To guide the segmentation, a second target known to localize to one of the structures can be imaged in a sequential session, contributing an additional color. Here we imaged Tom20, a mitochondrial outer membrane protein (**Fig 3b**). Since Tom20 and ATP-synthase partially colocalize on mitochondria, (**Fig 3a and b** arrows) the colocalization could be used to separate the initial image into separate colors. ATP-synthase was identified as those molecules which partially colocalize with Tom20 in a semi-automated way using a custom written colocalization analysis software (see Methods) for the initial segmentation followed by visual inspection for confirmation and manual correction. Similarly, lysosomes were identified as those molecules which did not colocalize with Tom20. Therefore, all three structures could be segmented into three different colors (**Fig 3c**). We similarly extended this approach to five-color imaging using three mouse antibodies and two additional antibodies from different species (**Fig 3d**).

## Discussion

We demonstrate multi-color STORM imaging using a single fluorophore. Our simple strategy eliminates a large number of technical problems and enables cross-talk free, multi-color STORM with the best performing fluorophore. We also demonstrate how the same region of interest can be repeatedly and robustly located on multiple days or microscopes using a very simple approach of imaging reference points on the sample chamber (such as the corners). This capability is very important since it means that the sample can be removed from the microscope to carry out the sequential labelling steps off-stage. Off-stage labelling means that each target can be immunostained with its own optimal set of conditions (e.g. incubation times and temperatures). More importantly, the microscope is not tied up during the multiple immunostaining steps. This simple method for locating a given region of interest without the need for any special grid can be broadly applied to any situation where sequential imaging is needed, including correlative microscopy where different microscopy modalities are combined [34].

**Figure 4:**
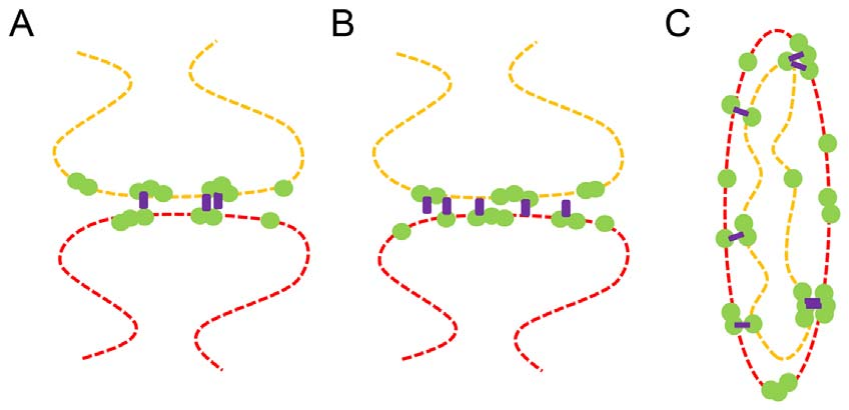
Conceptual application of multi-color STORM imaging using the same antibody species and same fluorophore. (A) Protein A (green) on the pre-synaptic structure (orange) and Protein B (green) on the post-synaptic structure (red) simultaneously colocalize with a third protein, Protein C (purple). (B) Protein C (purple) separately colocalizes with both Protein A (green) on the pre-synaptic structure (orange) and Protein B (green) on the post-synaptic structure (red) but rarely colocalizes with both proteins simultaneously. Sequential imaging using the same antibody species to label Protein A and B can be used to distinguish between these two scenarios (C) A second example following the same scenario as (A) and (B) but with mitochondrial inner and outer membrane proteins.

The ability to use the same fluorophore for multiple-colors also means that the extension of STORM to additional colors should only be restricted by the availability and performance of antibodies derived from distinct species. To this end, we also demonstrated that multiple targets can be imaged using the same antibody species and separated into multiple colors based on image segmentation. This approach provides additional flexibility for selecting antibodies for multicolor STORM, and is particularly important for STORM, since many of the antibodies for immunofluorescence which would have been acceptable for conventional microscopy methods do not produce a sufficiently high labelling density together with a sufficiently low background labelling for STORM. One example of a biologically-relevant scenario in which this approach would be useful is depicted in the conceptual drawing in **Figure 4**. One can imagine a scenario in which it would be interesting to know whether “nanoclusters” of a given protein (Protein C) simultaneously colocalize with “nanoclusters” of two other proteins (Protein A and Protein B) that localize to different structures (e.g. pre- and post-synaptic membranes or mitochondrial outer and inner membranes). If only one well-performing antibody species exists that can properly label Protein A and Protein B (e.g. mouse), then these two proteins can be labelled and imaged together using this one antibody species. If needed, a second antibody species can be used (e.g. chicken, rat, donkey etc…) to label one of the structures (e.g. the post synaptic structure or the outer membrane) and carry out guided segmentation of Protein A and B into separate colors. Finally Protein C can be labelled with yet a third antibody species and its simultaneous colocalization with both Proteins A and B can be determined. Labelling only Protein A and C or only Protein B and C, in two separate experiments would not allow discrimination between the scenario in which Protein C colocalizes with Protein A and B separately but not both of them simultaneously (**Fig 4a and b**).

Our approach builds upon a recently developed multiplexed version of point accumulation for imaging in nanoscale topography (PAINT) which uses a single fluorophore to achieve multi-color super-resolution imaging with labelled oligonucleotides [35]. The approach we present here is a simpler and cost-effective alternative as it relies on simple immunofluorescence labelling without the need for oligonucleotides and without the need for lengthy imaging sessions or long periods of microscope down-time. In combination, these approaches enable cross-talk free imaging of sub-cellular structures.

## Materials and Methods

### Mammalian Cell Culture

African green monkey kidney cells (BS-C-1, American Type Culture Collection, ATCC CCL-26) were maintained in culture using a complete growth medium (MEM Earle's with NEAA plus 10% FBS, 2 mM L-glutamine and 1 mM sodium pyruvate; a penicillin streptomycin mixture was added to prevent bacterial contaminations) at 37°C and 5% CO_2_. For the imaging experiments, Lab-Tek 1 coverglass chambers (NUNC) were coated with fiduciary markers (Nile Red fluorescence beads, Spherotech) and cells were seeded at a density of 20,000 to 50,000 cells per well.

### Sample Preparation

BS-C-1 cells were fixed with 37°C warmed fixation buffer (3% Paraformaldehyde (PFA), 0.1% Glutaraldehyde (GA) in PBS) for 10 minutes, then washed two times with 300 µL per well of PBS. The background fluorescence of GA was quenched by incubating the cells with 300 µL per well of 0.1% NaBH_4_ solution in PBS for 7 minutes at room temperature; finally the cells were washed three times with PBS. After fixation, the cells were incubated for 60 minutes at room temperature with blocking buffer [3% BSA (w/v), 0.2% TritonX-100 (v/v) in PBS]. For immunostaining, the cells were incubated for 50–60 minutes with the appropriate dilution of primary antibody in blocking buffer. Next, the cells were rinsed with washing buffer [0.2% BSA (w/v), 0.05% Triton X-100 (v/v)] twice (5 minutes each). Finally, the cells were incubated for 40–60 minutes with the appropriate dilutions of dye-labelled secondary antibodies, rinsed with washing buffer, and washed with PBS (5 minutes). Between imaging sessions, samples were incubated with 0.1% NaBH_4_ solution to quench the fluorescence of remaining fluorophores. Subsequent immunostainings were similar to the procedure described above, except the initial incubation with blocking buffer was reduced to 5 minutes. The primary antibodies used in these experiments were against: Tom20 (Santa Cruz Biotechnology, sc-11415), alpha tubulin (Abcam, ab6160), and LAMP2 (DSHB, H4B4). Appropriate secondary antibodies all containing the AlexaFluor405-AlexaFluor647 fluorophore pair were used.

### STORM Imaging

STORM imaging was carried out with either a custom-built microscope system as described previously [34] or with the Nikon NSTORM microscope both fitted with a 100X high NA (1.4 and 1.49 respectively) oil-immersion objective. Laser light at 647 nm (1 kW/cm^2^) was used for exciting AlexaFluor647 and switching it to the dark state and 405 nm laser light (up to ∼20 W/cm^2^) was used for re-activating the AlexaFluor647 fluorescence via an activator dye (AlexaFluor405)-facilitated manner. The emitted light from AlexaFluor647 was collected by the objective, filtered by an emission filter (ET705/72m), and imaged onto an EM-CCD camera at a frame rate of 50 Hz. Traditional dual color imaging was performed with two sets of secondary antibodies labelled with the same reporter dye (AlexaFluor647) but two different activator dyes (AlexaFluor405 and Cy3). In addition to the 405 nm laser light, an additional imaging cycle with 561 nm laser light as the activating light pulse was used for reactivating AlexaFluor647 linked to the second activator dye (Cy3).

*STORM Data Analysis*: STORM images were analyzed and rendered as previously described [34] using custom-written software and Insight3- kindly provided by Dr. Bo Huang. Briefly, peaks in single molecule images were identified based on a threshold and fit to a Gaussian to determine the *x* and *y* positions. The final images were rendered by using a Gaussian with a width that corresponds to the determined localization precision. Drift was corrected by correlating images generated from subsets of frames. The procedure was repeated for each color that was imaged, and then molecule lists from all of the imaging sessions were combined and rendered. This resulted in a slightly-misaligned multi-color image. To precisely align the different colors, the fiduciary markers were used, which were recorded alongside the raw STORM data and localized alongside the other molecules to generate high-resolution images of each fiduciary marker. These marker positions were used to generate a transformation matrix that aligns one image onto the other one (see Evaluation of Registration Error). The first imaging session was used as a reference and the subsequent imaging sessions were aligned to the first image. For some images, multiple primary antibodies from the same species were used. The resulting images were segmented based on colocalization which was detected using both a custom-written software as well as manually. The custom-written software determines colocalization as follows. The localizations of a particular channel are rendered at a resolution in which each pixel is 10×10 nm^2^ (i.e., the precision of our image alignment). A binary image is generated from this rendered image such that every pixel in the binary image that contains a localization gets a value of 1 and every pixel that does not contain a localization gets a value of 0. A similar binary image is also generated for an additional channel of interest. A pixel-wise logical AND operation is performed between these two binary images to generate a colocalized binary mask. Additional image morphology techniques (such as opening, closing and hole-filling) can also be applied to this binary mask. The pixels in this binary mask that have a value of 1 represent pixels where colocalization occurs and the localizations for each channel that are within these pixels are extracted to render a high-resolution colocalized image. The colocalized images were further examined and any false assignments were corrected manually. Alternately, high-resolution images were rendered and displayed in ImageJ. Areas of colocalization were delineated manually and then another custom-written software was used to sort the localizations inside each molecule list based on whether or not they were inside the areas of colocalization. After molecule lists were split using this guided segmentation approach, all molecule lists were recombined and used to render a high-resolution STORM image with each list displayed as a separate color in Insight3. For traditional multicolor imaging, each peak was color coded based on whether the emission was recorded immediately after 405 nm or 532 nm activation cycle. The peaks coming from frames which did not immediately follow an activation frame were discarded from the analysis.

### Evaluation of the registration error

A thousand-frame movie of fiduciary markers (fluorescent beads) was recorded. Next, the sample was taken off the stage, the same region of interest was relocated and a second thousand-frame movie of the beads was recorded. In addition, the same fiduciary markers were imaged using two different filter sets (set 1: ZT660 dichroic and ET705/72 emission filter; set 2: ZT488/561/642RPC dichroic and ET525/50 emission filter). The position of the beads was identified in each frame, leading to a small cluster of bead positions. The centroid cluster positions of the corresponding bead localizations in the two images were used for computing a transformation function. For choosing optimal beads for the registration the total perimeter of the polygon (a triangle in the case of three beads) enclosed by different bead combinations was computed and the beads that gave the largest perimeter were chosen. This approach enabled one to choose beads that were evenly distributed and covered a large range across the field of view. Two registration algorithms were tested by using a varying number of beads to carry out the registration (n = 3 to n = 10 beads). The first algorithm used a first-order polynomial affine transformation while the second algorithm used a second order polynomial local weighted mean transformation (the ‘cp2tform’ function of Matlab was used for each algorithm). The registration error was defined as the average distance between the transformed centroid positions from one movie with the centroid positions from the other movie (Equation 1):
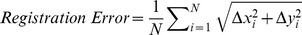
(Equation1)where Δ*x_i_* is the shift between the x-centroid positions of the *i*th bead in the two images and Δ*y_i_* is the shift between the y-centroid positions of the *i*th bead in the two images.

## Supporting Information

Figure S1 Determination of registration error. Registration error, calculated as the average distance between the transformed centroid positions of fiduciary markers from one image with the centroid positions from the other image. Two registration algorithms (first order polynomial affine and second order polynomial local weighted mean) were compared between sequential labeling (sequential) and traditional multi-color imaging using spectrally separated color channels (traditional). The boxes represent the 25th and 75th percentiles, the squares represent the mean, the lines represent the median and the whiskers represent the standard deviation.
(PNG)

Figure S2 Determination of color cross-talk in sequential and traditional multi-color imaging. Fluorophores are effectively quenched in between sequential imaging sessions. STORM image of microtubules (green) and mitochondria (magenta) recorded using sequential imaging (A) or traditional multi-color imaging with activator-reporter pairs (B). Regions of interest (ROIs) were drawn in both images around the microtubule structure (white) or around the background that excluded both microtubule and mitochondria structures (red). The localization density inside the white ROI in the second image (mitochondria) is a measure of the color cross-talk from the first into the second image. The localization density inside the red ROI in the second image is a measure of the background due to non-specific labeling. Scale bars 1 µm.
(TIF)

Figure S3 Effect of multiple washing steps on subcellular structural integrity. Microtubule structure is preserved after multiple washing steps. (A) Microtubules were labeled and imaged by recording a sufficient number of frames to reconstruct an image of the microtubules without exhausting all the fluorophores (red). The sample was then taken off the stage and five immunostainings were simulated by carrying out all the washing and incubation steps with blocking and washing buffers without actually adding antibodies. The same cell was relocated and imaged once again (green). Scale bars 2 µm (B) Zooms of the regions enclosed by the red squares in (A) are shown. Scale bars 500 nm (C) Zoomed-in regions of the rendered positions of two fiduciary beads enclosed by the white squares in (A) are shown. Scale bars 100 nm.
(TIF)

Table S1 Registration error for multiple sequential imaging of the same field of view. The sample was repositioned and the same field of view was imaged multiple times. The first image (Image 1) was used as a reference image and all subsequent images (Image 2- Image 4) were registered to this reference by using a first order polynomial affine transformation. Registration error was computed as the average distance between the centroid positions of fiduciary markers in different combinations of two sets of images (Image 1-Image 2, Image 1-Image3, Image 1-Image 4, Image 2-Image 3, Image 2-Image 4 and Image 3-Image 4). The registration error was not affected by the multiple repositioning of the same sample.
(DOCX)
