# Correction: Cross-Talk-Free Multi-Color STORM Imaging Using a Single Fluorophore

**DOI:** 10.1371/journal.pone.0111878

**Published:** 2014-10-22

**Authors:** The PLOS ONE Staff

In the Results section, there is an error in the first equation of the subsection titled “Sequential imaging using a virtual grid to repeatedly locate a given region of interest.” Please view the complete, corrected equation here: 
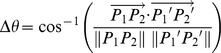



There are errors in the legend for Figure 2, “Multi-color STORM imaging using a single fluorophore.” Please see the complete, corrected Figure 2 here.

**Figure 2:**
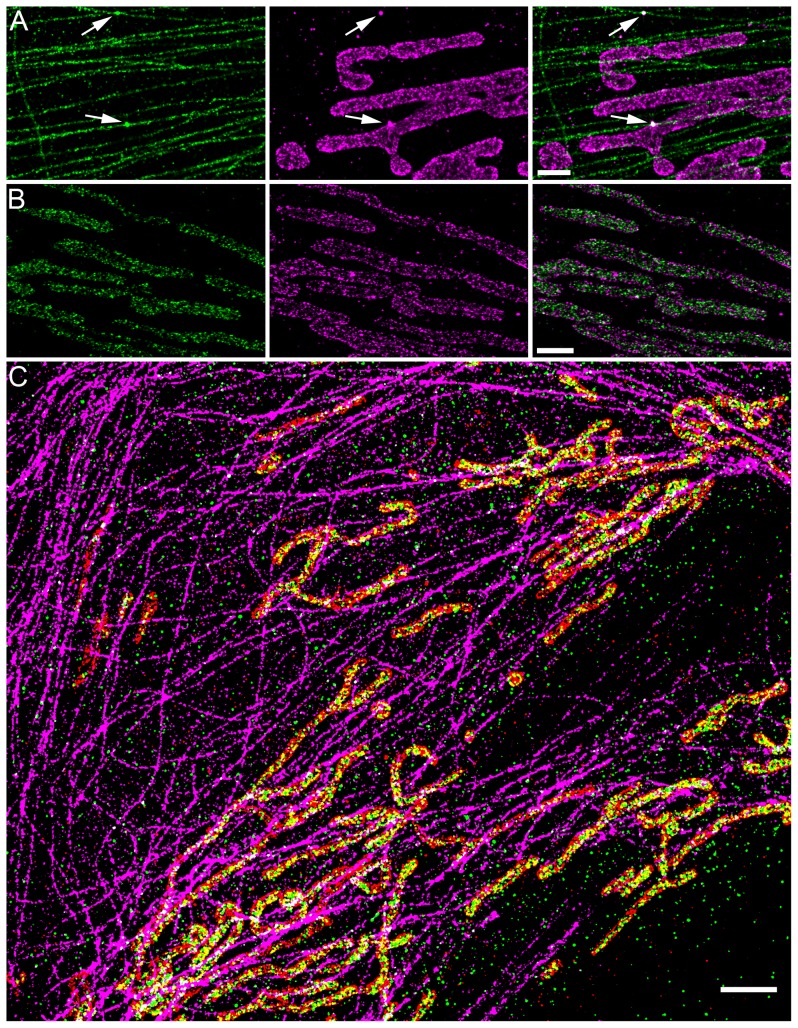
Multi-color STORM imaging using a single fluorophore. (A) Microtubules (green) and mitochondrial outer membrane protein Tom20 (magenta) imaged sequentially using the same fluorophore activator-reporter pair (AlexaFluor405-AlexaFluor647). Arrows show the localized positions of fiduciary markers (fluorescent beads) that were used for image alignment. (B) Mitochondrial outer membrane protein Tom20 (magenta) and inner membrane protein ATP Synthase (green). (C) Three-color image of microtubules (magenta), mitochondrial outer membrane protein Tom20 (red) and mitochondrial inner membrane protein (ATP-synthase, green) imaged sequentially using the same fluorophore activator-reporter pair (AlexaFluor405-AlexaFluor647). The discontinuous appearance of microtubules is due to the fact that we have used an anti-GFP antibody to label the GFP-α-tubulin and the endogenous α-tubulin is unlabelled in this scheme. The anti-GFP antibody was used since it offers a different antibody species to those used for ATP-synthase and Tom20. Scale bars, 1 µm (A–B), and 2 µm (C).
