# Supplementary figures and images for: Cross-Talk-Free Multi-Color STORM Imaging Using a Single Fluorophore
Source: PLoS One. 2014 Jul 7;9(7):e101772. doi: 10.1371/journal.pone.0101772 (PMC4084994; doi:10.1371/journal.pone.0101772)

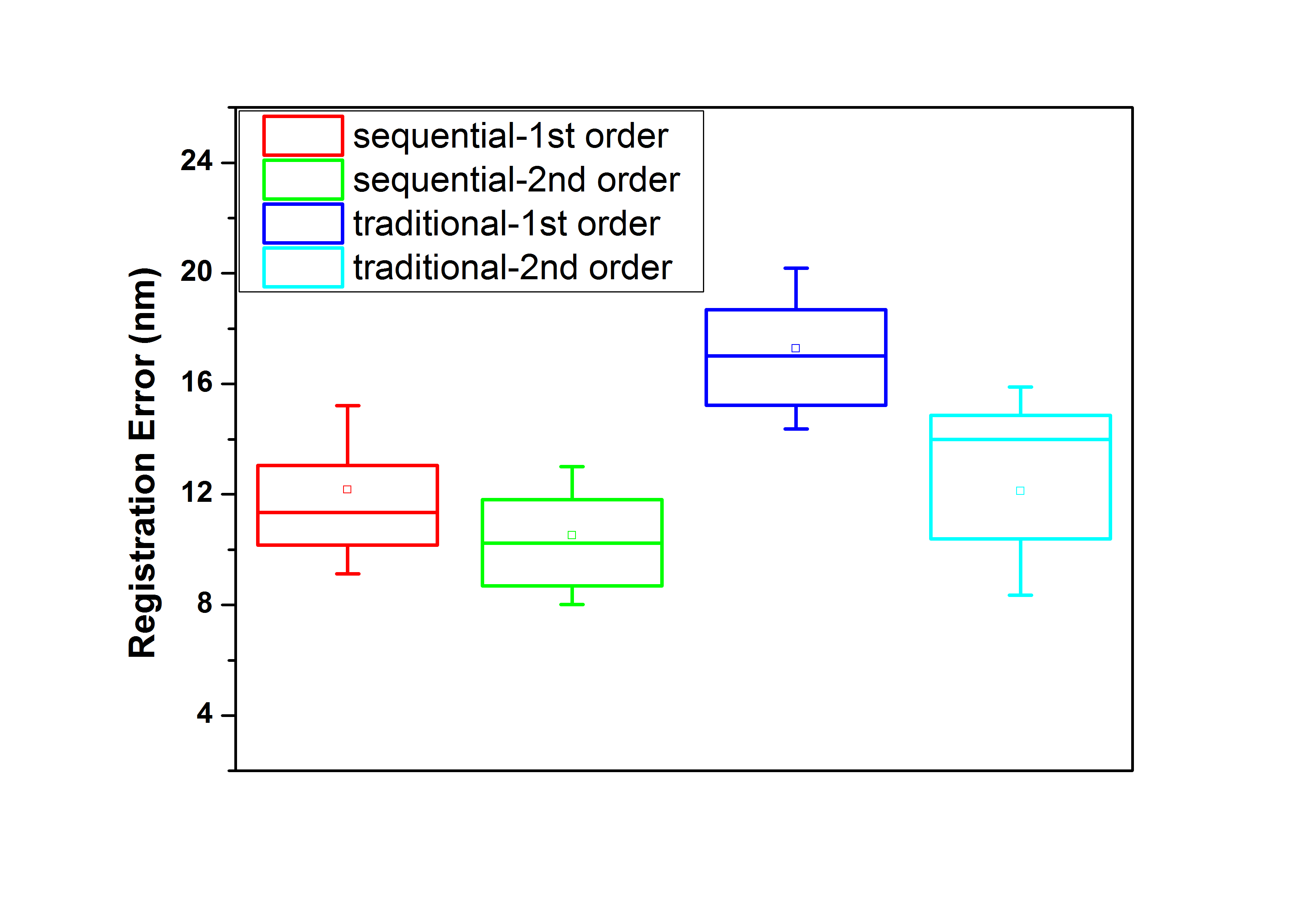

Supplement: Figure S1 — Determination of registration error. Registration error, calculated as the average distance between the transformed centroid positions of fiduciary markers from one image with the centroid positions from the other image. Two registration algorithms (first order polynomial affine and second order polynomial local weighted mean) were compared between sequential labeling (sequential) and traditional multi-color imaging using spectrally separated color channels (traditional). The boxes represent the 25th and 75th percentiles, the squares represent the mean, the lines represent the median and the whiskers represent the standard deviation. (PNG) [file pone.0101772.s001.png]

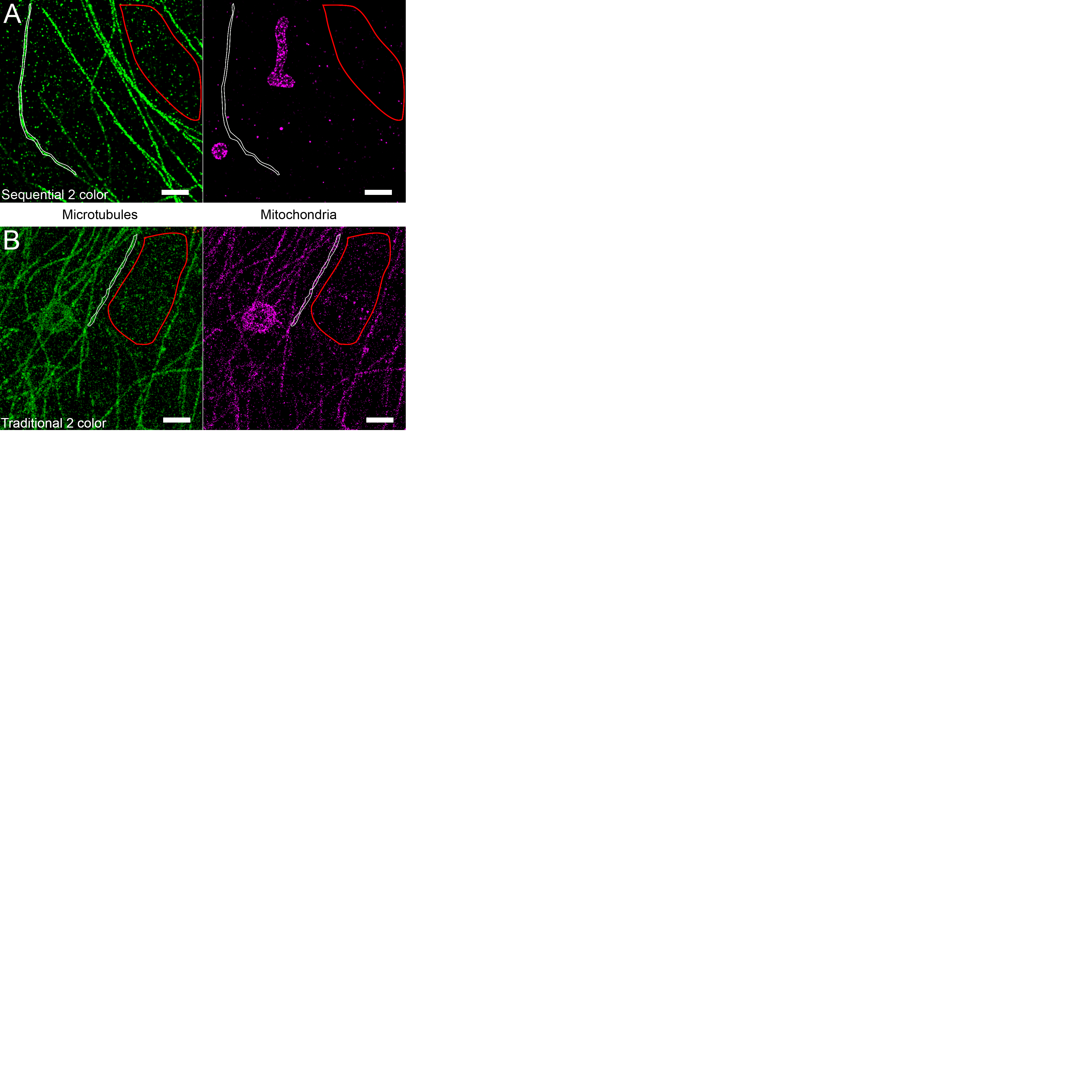

Supplement: Figure S2 — Determination of color cross-talk in sequential and traditional multi-color imaging. Fluorophores are effectively quenched in between sequential imaging sessions. STORM image of microtubules (green) and mitochondria (magenta) recorded using sequential imaging (A) or traditional multi-color imaging with activator-reporter pairs (B). Regions of interest (ROIs) were drawn in both images around the microtubule structure (white) or around the background that excluded both microtubule and mitochondria structures (red). The localization density inside the white ROI in the second image (mitochondria) is a measure of the color cross-talk from the first into the second image. The localization density inside the red ROI in the second image is a measure of the background due to non-specific labeling. Scale bars 1 µm. (TIF) [file pone.0101772.s002.tif]

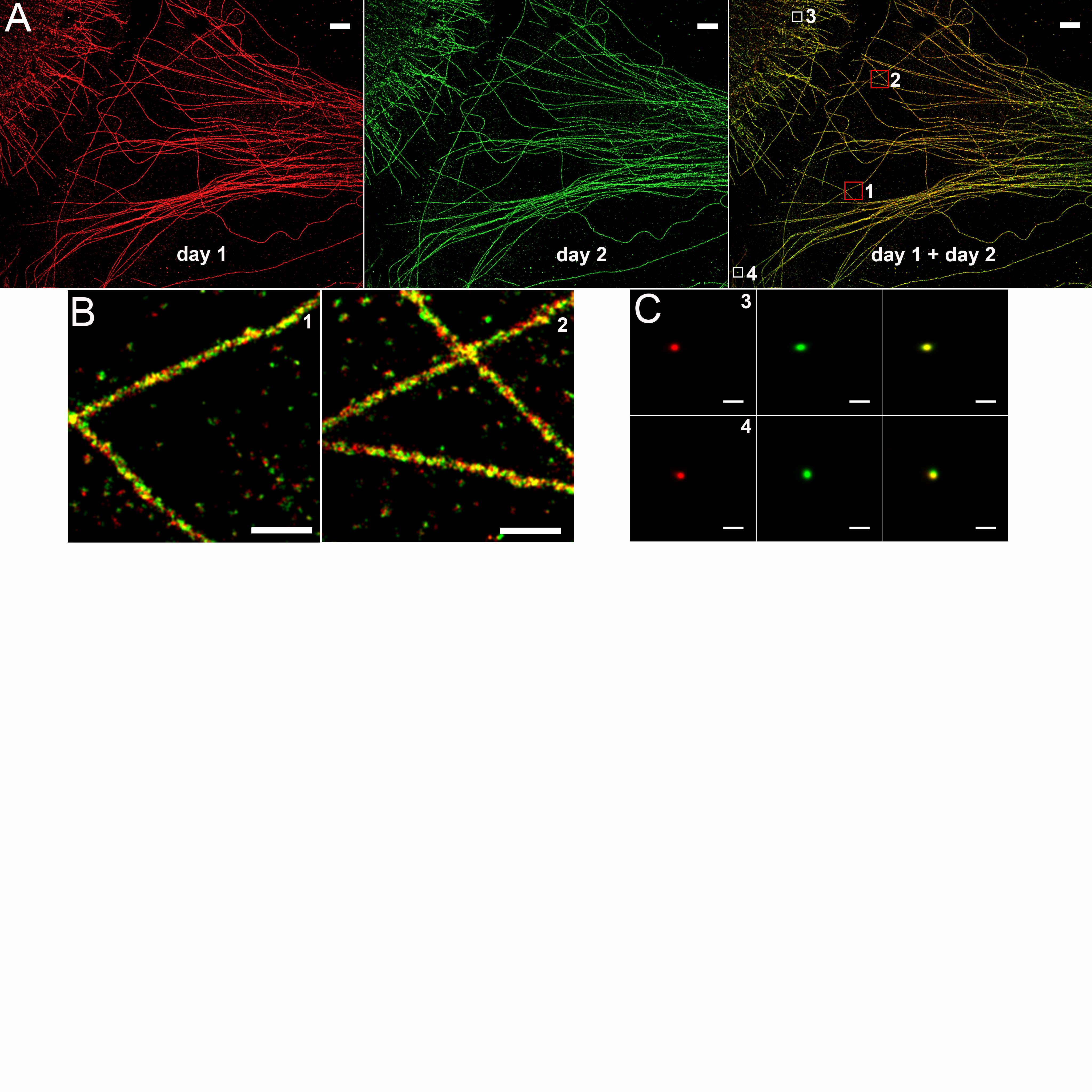

Supplement: Figure S3 — Effect of multiple washing steps on subcellular structural integrity. Microtubule structure is preserved after multiple washing steps. (A) Microtubules were labeled and imaged by recording a sufficient number of frames to reconstruct an image of the microtubules without exhausting all the fluorophores (red). The sample was then taken off the stage and five immunostainings were simulated by carrying out all the washing and incubation steps with blocking and washing buffers without actually adding antibodies. The same cell was relocated and imaged once again (green). Scale bars 2 µm (B) Zooms of the regions enclosed by the red squares in (A) are shown. Scale bars 500 nm (C) Zoomed-in regions of the rendered positions of two fiduciary beads enclosed by the white squares in (A) are shown. Scale bars 100 nm. (TIF) [file pone.0101772.s003.tif]
