# Supplementary material for: Cross-Talk-Free Multi-Color STORM Imaging Using a Single Fluorophore
Source: PLoS One. 2014 Jul 7;9(7):e101772. doi: 10.1371/journal.pone.0101772 (PMC4084994; doi:10.1371/journal.pone.0101772)
Supplement: Table S1 — Registration error for multiple sequential imaging of the same field of view. The sample was repositioned and the same field of view was imaged multiple times. The first image (Image 1) was used as a reference image and all subsequent images (Image 2- Image 4) were registered to this reference by using a first order polynomial affine transformation. Registration error was computed as the average distance between the centroid positions of fiduciary markers in different combinations of two sets of images (Image 1-Image 2, Image 1-Image3, Image 1-Image 4, Image 2-Image 3, Image 2-Image 4 and Image 3-Image 4). The registration error was not affected by the multiple repositioning of the same sample. (DOCX) [file pone.0101772.s004.docx]

|  | **Image 2** | **Image 3** | **Image 4** |
| --- | --- | --- | --- |
| **Image 1 (reference)** | 10.0 nm | 10.9 nm | 9.5 nm |
| **Image 2** |  | 10.7 nm | 9.8 nm |
| **Image 3** |  |  | 5.6 nm |
